# Temporal–Posterior Alpha Power in Resting-State Electroencephalography as a Potential Marker of Complex Childhood Trauma in Institutionalized Adolescents

**DOI:** 10.3390/brainsci14060584

**Published:** 2024-06-06

**Authors:** Gabriela Mariana Marcu, Ciprian Ionuț Băcilă, Ana-Maria Zăgrean

**Affiliations:** 1 Division of Physiology and Neuroscience, “Carol Davila” University of Medicine and Pharmacy, 020021 Bucharest, Romania; 2 Department of Psychology, Faculty of Social Sciences and Humanities, “Lucian Blaga” University of Sibiu, 550201 Sibiu, Romania; 3 Collective of Scientific Research in Neurosciences of the Clinical Psychiatry Hospital “Dr. Gheorghe Preda”, 550082 Sibiu, Romania; 4 Faculty of Medicine, “Lucian Blaga” University of Sibiu, 550169 Sibiu, Romania

**Keywords:** EEG marker, resting-state EEG, alpha waves, complex childhood trauma, adolescents, biomarkers, adversities

## Abstract

The present study explored whether, given the association of temporal alpha with fear circuitry (learning and conditioning), exposure to complex childhood trauma (CCT) is reflected in the temporal–posterior alpha power in resting-state electroencephalography (EEG) in complex trauma-exposed adolescents in a sample of 25 adolescents and similar controls aged 12–17 years. Both trauma and psychopathology were screened or assessed, and resting-state EEG was recorded following a preregistered protocol for data collection. Temporal–posterior alpha power, corresponding to the T5 and T6 electrode locations (international 10–20 system), was extracted from resting-state EEG in both eyes-open and eyes-closed conditions. We found that in the eyes-open condition, temporal–posterior alpha was significantly lower in adolescents exposed to CCT relative to healthy controls, suggesting that childhood trauma exposure may have a measurable impact on alpha oscillatory patterns. Our study highlights the importance of considering potential neural markers, such as temporal–posterior alpha power, to understanding the long-term consequences of CCT exposure in developmental samples, with possible important clinical implications in guiding neuroregulation interventions.

## 1. Introduction

Childhood trauma, particularly complex forms involving chronic interpersonal adversities such as parental negligence, has been linked to multiple domains of impairment in adolescence [1] and to mental health issues [2,3,4,5]. Electroencephalography (EEG) studies have suggested alterations in neural activity, including alpha oscillation abnormalities, in individuals with trauma-related disorders [2,6]. 

A certain topography of alpha oscillatory dysregulation in post-traumatic conditions would be of interest to both researchers and clinicians. The temporal lobes are believed to play a significant role in regulating emotions [7,8], given their functional connection to the emotion regulation pathways of structures such as the insular cortex, amygdala, and hippocampus [9]. Therefore, they may be good candidates for exploring EEG markers for fear regulation disorders. Although there have been some findings on the dysregulation of neural connectivity in these areas [10,11,12], the relationship between EEG oscillations, such as alpha power in temporal locations, and psychological trauma is still poorly understood.

The impact of complex childhood trauma (CCT) can be profound, particularly in children and adolescents. It can manifest as a range of cognitive and emotional difficulties, risky behaviors, and mental health challenges. Often, clinical trauma symptomatology is indistinguishable from other stress and emotion regulation disorders, making targeted treatment challenging. Also, children with clinically significant trauma symptoms in foster care are 46% more likely to experience placement instability, highlighting the need for early trauma assessment and intervention [13]. Therefore, having reliable neurophysiological patterns detectable as traces of CCT exposure could be important because complex trauma survivors often receive inaccurate diagnoses, such as borderline personality disorder (BPD), leading to stigma and ineffective treatment. Adolescents who reported multiple or chronic trauma events were more likely to exhibit symptoms associated with developmental trauma disorder (DTD), highlighting the need for improved diagnosis and treatment of complex trauma in youth [14]. The proper identification of CCT or the diagnosis of complex post-traumatic stress disorder (CPTSD) can lead to improved self-understanding, reduced stigma, and effective treatment choices.

The spectral distribution of EEG at the scalp level, which can be obtained through qEEG analysis, has been suggested as a neurophenotype. It has been proven to be stable in repeated measurements [15] and is associated with neural system functions that produce specific behaviors [16]. 

Here, we propose temporal–posterior alpha power as a potential EEG marker, as it indicates neural oscillatory activity in regions associated with emotion regulation and memory consolidation [17,18]. Previous studies have suggested alterations in EEG patterns among individuals with post-traumatic stress disorder (PTSD), including increased alpha activity, particularly in the right temporal lobe [11,19,20]. 

Alpha oscillations are typically associated with a state of cortical idling or inhibition, reflecting decreased neuronal activity in the regions in which they occur. In the right temporal or temporo-occipital sites, excessive alpha oscillations may indicate an overactive inhibitory mechanism, potentially leading to disruptions in sensory processing, attentional control, and emotional regulation, depending on which large-scale network is involved. Functionally, excess inhibition could manifest as difficulties in recognizing and interpreting visual or auditory stimuli, impairments in attentional focus or sustained attention, and challenges in regulating emotional responses to environmental stimuli [21,22]. 

The relationship between affective system dysregulation, neurotransmitters, and abnormalities in alpha oscillations has been explored in several studies, most of which are related to GABAergic neurotransmission, which is essential for generating alpha oscillations. Research suggests that GABAergic dysfunction may contribute to abnormalities in alpha oscillations observed in conditions such as anxiety disorders and PTSD [23,24].

In brain physiology, excessive power in alpha oscillations in the temporo-occipital regions can arise because of several factors related to underlying neural dynamics and connectivity. The thalamocortical dysrhythmia model of neuropsychiatric disorders [25] refers to a disruption in the normal oscillatory interactions between the thalamus and cortex, leading to excessive synchronized oscillations, including alpha rhythms. In this model, abnormal thalamic inputs to the cortex result in increased alpha power, particularly in regions receiving projections from the thalamus, such as the temporo-occipital cortex. These states have been conceptualized as oscillopathies [26] and may reflect malfunctioning networks. It has also been suggested that reduced neural excitability in the temporo-occipital region can lead to heightened synchronization and excessive alpha power [27]. Additionally, long-term neuroplastic changes in response to injury, environmental factors, or pathological conditions can reshape the neural circuitry and promote aberrant oscillatory patterns [28].

### Temporal Alpha and Fear Conditioning and Learnig

Evidence suggests a relationship between excessive alpha power in the temporal region and fear-related processes, including fear conditioning and fear learning circuits [20,29]. 

Fear conditioning is a type of associative learning in which a neutral stimulus is associated with a fearful event, leading to a conditioned fear response. This process involves complex neural circuits, including regions of the temporal lobe such as the amygdala, which play a crucial role in processing and encoding emotional memories [30]. The proposed neurobiological correlates of hypervigilance in PTSD may result from heightened salience processing [31], but may also impair self-awareness and autobiographical memory [32]. According to a meta-analysis conducted by Koch et al. in 2016, individuals with PTSD show hyperactivity in the ventral anterior cingulate cortex and parahippocampus/amygdala, as well as hypoactivity in the (posterior) insula and middle frontal gyrus. Additionally, functional connectivity studies indicate enhanced connectivity in the salience network, but decreased connectivity in the default mode network in PTSD adults [8,32] and altered resting-state brain connectivity patterns in PTSD adolescents, suggesting a common basis for intrusive trauma recollection and impaired episodic memory [33].

Few studies have reported changes in EEG patterns [34], including increased alpha power, in individuals with PTSD [35] or during fear conditioning tasks [29,35]. These changes may indicate a dysregulation of fear-learning circuits. Studies using resting-state MEG have identified abnormal neural activation in multiple cortical areas and medial temporal structures related to emotional processing in veterans with PTSD [8]. However, the specific role of excessive temporal alpha power in fear conditioning circuits is not fully understood. Alterations in alpha power could reflect diverse underlying neural processes related to emotional regulation [36], attentional biases [37], or other cognitive functions [38] implicated in PTSD and fear-related disorders. According to Heller’s neuropsychological model, separate neural systems underlie the valence and arousal dimensions of emotions, with a parietotemporal system modulating arousal in the right hemisphere. [39]. Despite indirect or anecdotal references pointing to increased alpha activity, particularly in the right temporal lobe in traumatized individuals, there is a lack of scientific research linking alterations in EEG temporal–posterior alpha patterns to PTSD. Clinicians applying neuroregulatory techniques through individualized protocols often refer to the “T6-alpha” as a biomarker for psychological trauma in their clinical communications; therefore, this potential marker requires systematic validation through scientific investigation.

To our knowledge, this is the first study to examine temporal–posterior alpha power in EEG as a potential marker of CCT in adolescents. Analyzing electric oscillations on the surface of the brain’s temporal lobes can aid in comprehending the neural mechanisms underlying trauma-related psychopathology, considering its association with fear circuitry, learning, and conditioning.

This study aimed to investigate the impact of complex childhood trauma exposure on temporal–posterior alpha power, assessed via resting-state EEG, in an institutionalized adolescent population.

We examined temporal–posterior alpha power in adolescents with a history of complex childhood trauma and compared it with that in healthy controls. Furthermore, we explored the association between temporal–posterior alpha power and self-reported symptoms of trauma-related psychopathology, including PTSD subscales, dissociation, and depression, in adolescents.

We hypothesized that adolescents exposed to CCT would exhibit alterations in temporal–posterior alpha power, characterized by either increased or decreased activity, compared to neurotypical matched controls. We preregistered our hypothesis and analytic approach at https://osf.io/5ya3v/?view_only=41270f74556745c399c0307579cb66d7 (accessed on 2 June 2024).

## 2. Materials and Methods

### 2.1. Participants/Data

The data for this study were collected as part of a larger project concerning EEG neuromarkers of trauma in adolescents (Marcu et al., under review). This project was reviewed and approved by institutions legally responsible for the participants (the National and Local Child Protection Agencies) and institutions involved in the study implementation (local university and psychiatric hospital). The full data collection protocol, which includes the inclusion/exclusion criteria and EEG data collection and processing procedures, was preregistered (prior to collecting any data) and can be found at https://osf.io/m3fdv/?view_only=cc6d743714504749a85cc093c85039cc (accessed on 2 June 2024). 

The target population was adolescents between 12 and 17 years of age, living in residential housing for a minimum of three consecutive months, with a history of one or more traumatic childhood experiences, without any suspected neurological pathologies such as epilepsy or cerebral palsy, and no changes in medication/psychotherapy 3 months prior to data collection. All necessary measures were implemented to ensure participant confidentiality and data protection.

The trauma group in the current study was established based on data from 26 adolescents (8 males, 18 females) with a history of neglect or abuse who had been placed in the foster care system in Romania. Informed consent was obtained from all participants and their legal guardians prior to study participation, and a compensation of 20 RON (~4 USD) was offered to each participant. Participants were screened for trauma exposure, trauma-related symptoms, and psychiatric comorbidities (depression, alexithymia, and dissociation) (for descriptive statistics and details of screening instruments, see Table 1).

The control group consisted of 28 adolescents and was established based on EEG data that are available on an open repository, the Child Mind Institute Biobank-Multimodal Resource for Studying Information Processing in the Developing Brain (MIPDB) [40].

**Table 1:** Descriptive statistics for the trauma and control groups.

	Group			
	Trauma		Control	
	M	SD	M	SD
Age	15	1.73	14.21	1.50
IQ ^a^	82.85	1.73	97.04	1.50
PTSD (screening) ^b^	17.5	9.5	Not available/Not applicable	
PTSD (assessment) ^c^	11.5	5.5		
CPTSD ^c^	20	8.8		
DSO ^c^	8.5	4.9		
Alexithymia ^d^	15.4	8.5		
Dissociation ^e^	2.2	2.2		
Depression ^f^	10.8	8.3		
Years in the protection system	9.6	4.8		
	Yes (n)	No (n)		
PTSD (criteria met)	13	12		
CPTSD (criteria met)	6	19		
n	26		28	

Note. The full description of this sample was also reported in Marcu et al. (under review) and the information in this table is reprinted with permission. Abbreviations: post-traumatic stress disorder (PTSD), complex post-traumatic stress disorder (CPTSD), disturbances in self-organization (DSO). ^a^ RAVEN for TG; WASI II for CG. ^b^ UCLA PTSD Reaction Index [41]. ^c^ International Trauma Questionnaire-Child and Adolescent (ITQ-CA) [42]. ^d^ Children’s Alexithymia Measure [43]. ^e^ Adolescent Dissociative Checklist [44]. ^f^ Beck Depression Inventory II [45].

### 2.2. Procedure

EEG data were collected using a 19-channel Mitsar-EEG-BT Lite QEEG edition system along with a 21-electrode EEG cap (Medcap) featuring AgCl electrodes placed according to the 10–20 system, as described in the Preregistration for Data Collection (https://osf.io/s2h58/?view_only=6bacd53d6dee4d7c9b9c45e0b48978bb (accessed on 2 June 2024)). The participants completed a resting-state EEG recording while seated comfortably with their eyes open and then closed for a designated duration (5 min for each condition). The EEG data were then processed and analyzed to extract the temporal–posterior alpha power within the frequency range of 8–12 Hz using the EEGLab GUI [46]. The preprocessing pipeline involved bandpass filtering (0.1–40 Hz), bad channel removal, visual inspection for artifact removal, resampling to 250 Hz, re-referencing to average, and Independent Component Analysis (ICA) decomposition for ocular and muscle artifact correction (IClabel plugin). No bad channels were removed before analysis. Epoching and frequency analysis were performed using the EEGLAB Spectral Analysis plugin (eegstats ver. 1.2) to compute the power spectral density (PSD) of the EEG data at temporal–parietal locations (T5 and T6). This step involves applying a Fast Fourier Transform (FFT) spectral analysis technique to estimate the power spectrum (2 s epochs, 1s overlap, at a frequency resolution of 0.5 Hz).

### 2.3. Data Analysis

For each resting-state condition (eyes open (EO), eyes closed (EC)), we used a 2 between (group: trauma vs. control) × 2 within (location: left vs. right) mixed analysis of variance to examine our preregistered hypothesis (dependent variable: temporal–posterior alpha power). We did not preregister planned contrasts; therefore, we ran pairwise comparisons instead and applied a false discovery rate correction [47]. However, of theoretical interest are the comparisons between groups for each location (left, right), which we report here. The same analyses were run first with participant age (preregistered) and then with IQ (exploratory), each added as covariate. For exploratory purposes, for the trauma group, we also computed the Pearson correlation coefficient between the temporal–posterior alpha power for each condition (EO, EC) and each location (left, right) and participants’ phenotype scores (trauma-related symptoms and psychiatric comorbidities). 

All statistical analyses were run using R, version 4.2.3 (R Core Team, 2023). The data used in this study, R code, and full output for analyses are available at: https://osf.io/kf4x5/ (accessed on 2 June 2024). 

## 3. Results

**Figure 1:**
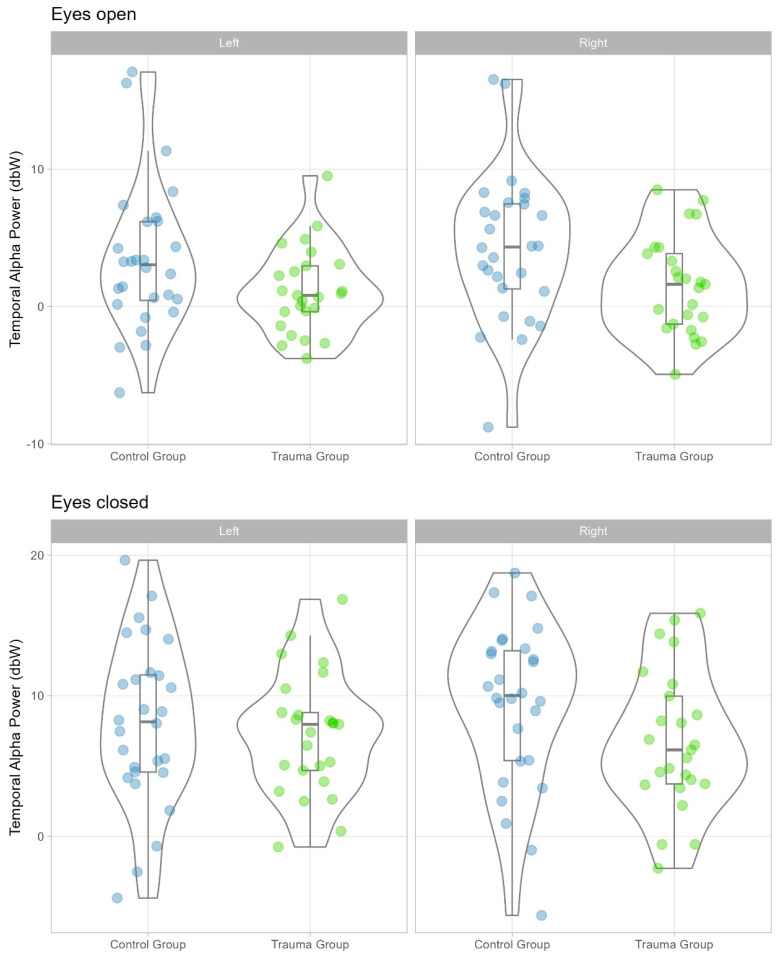
Distribution of temporal-posterior alpha power values by group, location, and condition. Green dots = Trauma Group, Blue dots = Control Group.

The distribution of temporal–posterior alpha power values for the trauma group and the control group, by condition (eyes-open, eyes-closed) and by location (left, right), is displayed in Figure 1. 

We found significant differences in temporal–posterior alpha power between the trauma group and the control group only in the EO condition. Specifically, there was a significant main effect of group, F(1, 51) = 4.40, *p* = 0.041, η^2^p = 0.08, 90% CI (0.00, 0.22), η^2^G = 0.08. The pairwise comparison showed that temporal–posterior alpha power was lower for the trauma group, M = 1.34, SE = 0.87, relative to the control group, M = 3.86, SE = 0.82, t (51) = −2.52, *p* = 0.041, Cohen’s d = −1.12, 95% CI (−2.19, −0.05). The main effect of location (left, right) was not statistically significant, F(1, 51) = 3.87, *p* = 0.055, η^2^p = 0.07, 90% CI (0.00, 0.21.), η^2^G = 0.01. The interaction between group and location was also not statistically significant, F(1, 51) = 0.53, *p* = 0.055, η^2^p = 0.07, 90% CI (0.00, 0.21), η^2^G = 0.01.

In the eyes-closed condition, there was a statistically significant interaction effect between group and location, F(1, 51) = 4.15, *p* = 0.047, η^2^p = 0.08, 90% CI (0.00, 0.21), η^2^G = 0.01. Pairwise contrasts, however, were not statistically significant (all *p*s > 0.234). There was no significant main effect of group, F(1, 51) = 1.48, *p* = 0.229, η^2^p = 0.03, 90% CI (0.00, 0.14), η^2^G = 0.03, and no significant main effect of location, F(1, 51) = 0.76, *p* = 0.386, η^2^p = 0.02, 90% CI (0.00, 0.11), η^2^G = 0.00. 

Adding age as a preregistered exploratory covariate suppressed the effect of group we observed in the eyes-open condition and the interaction we found in the eyes-closed condition. Adding IQ as an exploratory covariate showed that it had a significant effect on temporal alpha, but since the analysis was not preregistered and the sample is small, no valid conclusion could be drawn from this analysis in the present study (see full analysis output on the OSF). There were also no statistically significant correlations between temporal–posterior alpha and phenotype scores in the trauma group.

**Figure 2:**
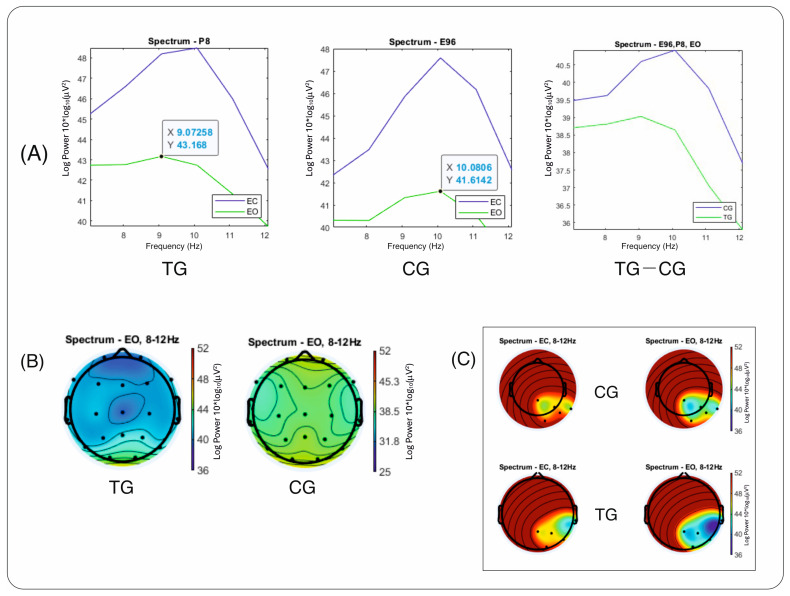
(**A**) Right temporal–posterior channel spectral graph of RMS power for conventional alpha bandwidth (8–12 Hz) in the trauma group (TG) (left), control group (CG) (middle), and in both groups (right). (**B**) Topoplots of the spectral distribution of alpha waves in CG and TG. (**C**) Topographical map of alpha waves at selected electrode sites (T6 and neighboring sites T4, P4, Pz, and O2) in both CG (top) and TG (bottom).

The results that distinguish the TG (trauma group) from the CG (control group) in temporal–posterior alpha in the EO condition are presented as spectral graphs and maps in Figure 2. The RMS power of the right temporal channel—the T6 channel from the 10–20 nomenclature, output as P8 of the 10-10 system in the TG, and its correspondent E96 (128 channel system) in the CG—is plotted for both groups (Figure 2A). The graph shows a lower PSD of alpha in the temporal–parietal site for the TG. It also shows a two-peak alpha wave in the TG. In addition, the topography of alpha distribution across the scalp is presented for the two groups, TG and CG (Figure 2B), indicating that there is a visible deficit of temporal–posterior alpha in the TG compared to the CG in the same power range. The topographical map shows the absolute power of brain waves across the scalp in the 8–12 Hz frequency range, with a restrained distribution and generally lower in the TG group, consistent with other findings on low EEG power in traumatized adolescents (Marcu et al., manuscript under review). When a specific region of interest consisting of five electrode sites (T6 and its neighboring sites T4, P4, Pz, and O2) was selected for averaging power, a noticeable difference was observed between the groups (Figure 2C). In this region, the TG shows a more widespread alpha area and also lower spectral alpha values for the right temporal site. Further spectral maps of absolute and relative EEG power and topographical maps compared with those of matched controls from the HBI Database are available in the Supplementary Materials.

## 4. Discussion

As hypothesized, we found significant differences in temporal alpha power between the trauma and control groups, with dissociation depending on the resting-state condition (eyes-open, eyes-closed). Particularly, we found that in the eyes-open condition, temporal alpha was lower in both the right and left hemispheres in adolescents with trauma relative to healthy controls. In the eyes-closed condition, the power of right temporal alpha was lower in adolescents with trauma compared to healthy controls, but this difference was not statistically significant. However, the effect size of this difference might be worth replicating in larger samples to determine whether the right hemisphere is most reactive to trauma exposure. 

Our findings suggest that childhood trauma exposure may have a measurable impact on alpha oscillatory patterns, specifically in the EO condition. This result might be interpreted as a higher cortical activation of temporal–parietal areas, which could be explored further in connection to symptoms of PTSD, such as re-experiencing and hyperarousal/threat-related dysregulation. However, adding age as a preregistered exploratory covariate suppressed the effect of group we observed in the EO condition, suggesting that alpha power might be an EEG feature that is not very stable with age. This finding aligns with recent studies on infants, which have shown that nonlinear EEG measures like fractal dimension, entropy, and connectivity features remain relatively stable in a healthy infant population during development, while the power of frequency bands is much less stable [48]. The age-related differences in the power of frequency bands may also account for the variations in temporal–parietal alpha power between traumatized adolescents and adults compared to healthy controls.

It is important to interpret these results within the broader framework of other EEG, cognitive, and behavioral characteristics that are associated with the aftermath of childhood trauma. We are referring here to cognitive developmental impairments, characterized by altered executive functioning [49,50] and lower IQ or cognitive deficits [51,52], and also to qEEG features like iAPF, posterior alpha power, alpha asymmetry, or total EEG power (Marcu et al., in review). Our findings show that IQ may play a moderating role in the relationship between CCT and EEG alpha power in temporal–parietal sites. This might be related to the relationship between alpha rhythm variants and intelligence/cognitive deficits [53,54], and it supports the existing literature on the impact of trauma on neurodevelopment [55,56]. However, it is important to note that this result may be limited because the IQ assessment of the two groups was conducted using two different instruments, which are otherwise correlated [57,58]: the trauma group was assessed with the Raven test, while the controls were assessed with the WASI II.

It is worth noting that the sources of information about alpha power in the temporal–parietal region of the brain being linked to psychological trauma in resting EEG scans, particularly in the right hemisphere, are mostly from clinical or scientific conferences/training settings and less from academic papers. Moreover, this proposed marker has mainly been observed or reported in traumatized adults who have completed brain development and who have had a longer time period since the traumatic experience. This allowed for the occurrence of both positive phenomena, such as coping strategies, resilience, or post-traumatic growth, and of negative ones, such as mental health issues like depression, addiction, or borderline personality disorder, which may also impact the EEG. Also, the alpha power marker for PTSD, especially in posterior regions, was found to show inconsistent results in an extensive review of PTSD predicting EEG biomarkers [59], which concluded that more studies are needed for this marker. Our research suggests that lower alpha power in the measured brain region appears to be associated with trauma in adolescents. These results align with the findings of other studies in the literature [60,61].

Studies have shown that individuals with a history of childhood trauma exhibit alterations in EEG patterns, particularly in alpha rhythms. For instance, research has highlighted an inverse relationship between childhood abuse and alpha power [62], negative correlations between neglect and alpha power at left parietal electrodes in resting conditions [12], and greater right asymmetry in maltreated individuals [63,64,65]. EEG studies have a limitation in that they reflect only the surface electrical activity without providing information about possible generators and their locations. To address this limitation, EEG research has been complemented with other imaging techniques such as fMRI. This combination has helped to solve the EEG inverse problem, which has revealed that childhood trauma is associated with impairments in neuronal networks involved in emotional expression and regulation, threat detection, arousal, information processing, and sensory gating [59,66]. To gain a more comprehensive understanding of this, a multimodal investigation of childhood trauma-related phenomena can provide further insight into the development of a common hypothesis based on alpha oscillatory activity.

Aside from the main findings, our observations revealed a double alpha peak in the trauma group, which could possibly be interpreted as being generated by two or more independent generators from different regions of the brain. As the peaks do not emerge near the ends of the frequency band, this was not considered a ringing artifact (Gibbs phenomenon) of spectrum leakage [67]. It is beyond the scope of this paper to further investigate the sources’ localization, but this observation might be of interest for more extensive research on generators of alpha rhythms in traumatized individuals.

While our findings contribute to the growing body of literature on neurobiological markers of CCT, several limitations should be acknowledged. First, the sample size was relatively small, which may limit the generalizability of the findings. Future research with larger, more diverse samples is needed to confirm and extend our findings. Also, the self-report measures may be subject to biases and inaccuracies, particularly in retrospective reporting of traumatic experiences.

Nevertheless, the current study is part of a larger effort by many researchers to gain a deeper understanding of the neuropsychological aftermath of CCT in developmental samples. Research suggests that current diagnostic criteria for PTSD/CPTSD may not adequately identify how adolescents express PTSD symptoms in response to childhood and adolescent complex trauma [68,69]. A developmental trauma framework was proposed to offer a broader perspective on the mental health of adolescents affected by CCT [70,71]. This framework highlights the importance of considering symptoms to be reactions to trauma rather than as separate mental health issues. The complex trauma framework [52] provides a strength-based, survival-driven reframe of trauma, considering an individual’s behaviors, interpersonal difficulties, identity and self-image, and psychiatric diagnoses as adaptive strategies to survive overwhelming experiences in a hostile world [72]. However, while some adolescents with CCT may show high internalizing symptoms or dissociation scales in response to trauma, others may appear less affected and score lower or “normal” on trauma or psychopathology assessments. This raises the question of how we should measure CCT and raises the importance of validating EEG screening markers such as the one presented here.

The present study focused on temporal alpha power as a potential biomarker of CCT exposure in adolescents. Future research should explore other neural markers of CCT, especially the temporal dynamics of brain oscillations and event-related potentials, and examine their collective utility in predicting trauma-related psychopathology. Furthermore, integrating peripheral physiological measures and electroencephalography (EEG) with comprehensive clinical assessments may provide a more nuanced understanding of the impact of CCT.

## 5. Conclusions

In conclusion, our study highlights the importance of considering neurophysiology, such as resting-state EEG power in the alpha band, in understanding the long-term consequences of CCT exposure and in the early detection of CCT exposure in developmental samples. Thus, more effective interventions could be developed to mitigate the impact of CCT on affected individuals.

We anticipate that our findings will further contribute to the development of a comprehensive predictive model of CCT’s impact, tailored to developmental samples. Such a complex model, which takes into account neurophysiological and psychological characteristics, can be a valuable tool for clinical screening, with high specificity in classifying traumatic exposure. 

The identification and validation of more neurophysiological markers—like posterior alpha power, parietal theta power or total EEG power-, may better inform the development of targeted interventions for trauma-related psychopathology in adolescents. For example, for adolescents in residential centers, they may contribute to tailoring interventional programs to improve functionality and help the trauma-affected adolescents to form secure attachment and stable relationships while reducing trauma symptoms and risky behaviors. Potential clinical implications may include using an EEG-based predictive model for early identification and intervention in larger youth populations with CCT, such as personalized qEEG-guided neurofeedback protocol development.

## Data Availability

The data used in this study, R code, and full output for analyses are available at https://osf.io/kf4x5/?view_only=af16fc85ece94b1cb503ac93b6a68dd0 (accessed on 2 June 2024).

## Supplementary Materials

The following supporting information can be downloaded at: https://www.mdpi.com/article/10.3390/brainsci14060584/s1, Figure S1: Distribution of temporal—posterior alpha power; Figure S2: Spectral Graphs and Maps, Table S1: Sample Descriptives;
