# Supplementary material for: Temporal–Posterior Alpha Power in Resting-State Electroencephalography as a Potential Marker of Complex Childhood Trauma in Institutionalized Adolescents
Source: Brain Sci. 2024 Jun 6;14(6):584. doi: 10.3390/brainsci14060584 (PMC11201643; doi:10.3390/brainsci14060584)
Supplement: Supplementary file 1 [file brainsci-14-00584-s001.zip › Figure S1 Distribution of temporal ¿C posterior alpha power.pdf]

Figure S1: Distribution of temporal – posterior alpha power

Selected electrodes of interest: T4, T6, O2,P4, C4

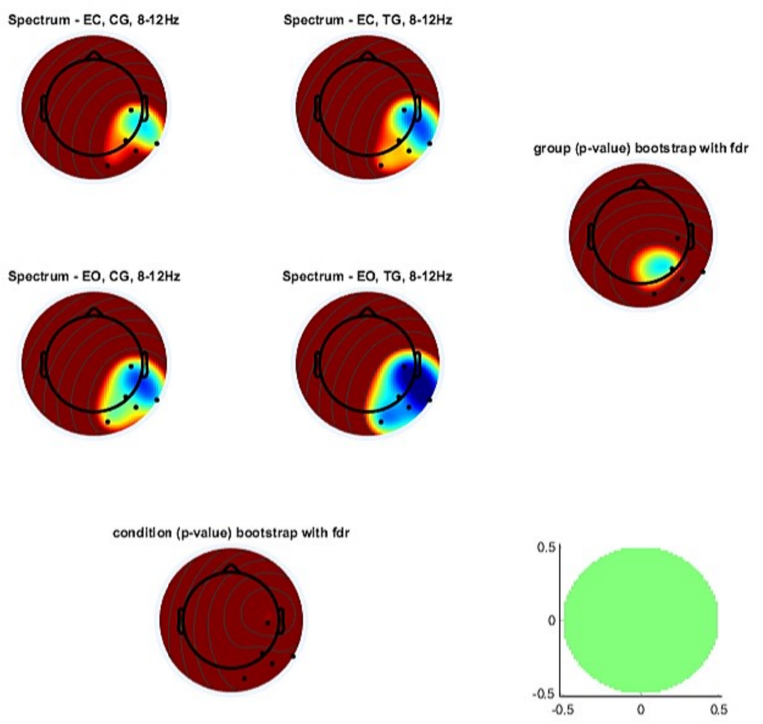

Note: interaction group * condition with
FDR correction for multiple comparisons.

EO = eyes open
EC = eyes closed
CG = control group
TG = trauma groups
