# Supplementary material for: Temporal–Posterior Alpha Power in Resting-State Electroencephalography as a Potential Marker of Complex Childhood Trauma in Institutionalized Adolescents
Source: Brain Sci. 2024 Jun 6;14(6):584. doi: 10.3390/brainsci14060584 (PMC11201643; doi:10.3390/brainsci14060584)
Supplement: Supplementary file 1 [file brainsci-14-00584-s001.zip › Figure S2 Spectral graphs and maps.pdf]

Supplementary Material – Spectral graphs and maps

* Analyses made with WinEEG Software version 3.13.26 (2024)

Trauma Group (TG) – Eyes Closed Condition

25 participants with Complex Childhood Trauma (CCT)

Graphs of EEG absolute power spectra

Fragment: Grand Average, Offset: 0.00 s, Length: 314.08 s, Number of epochs 20.

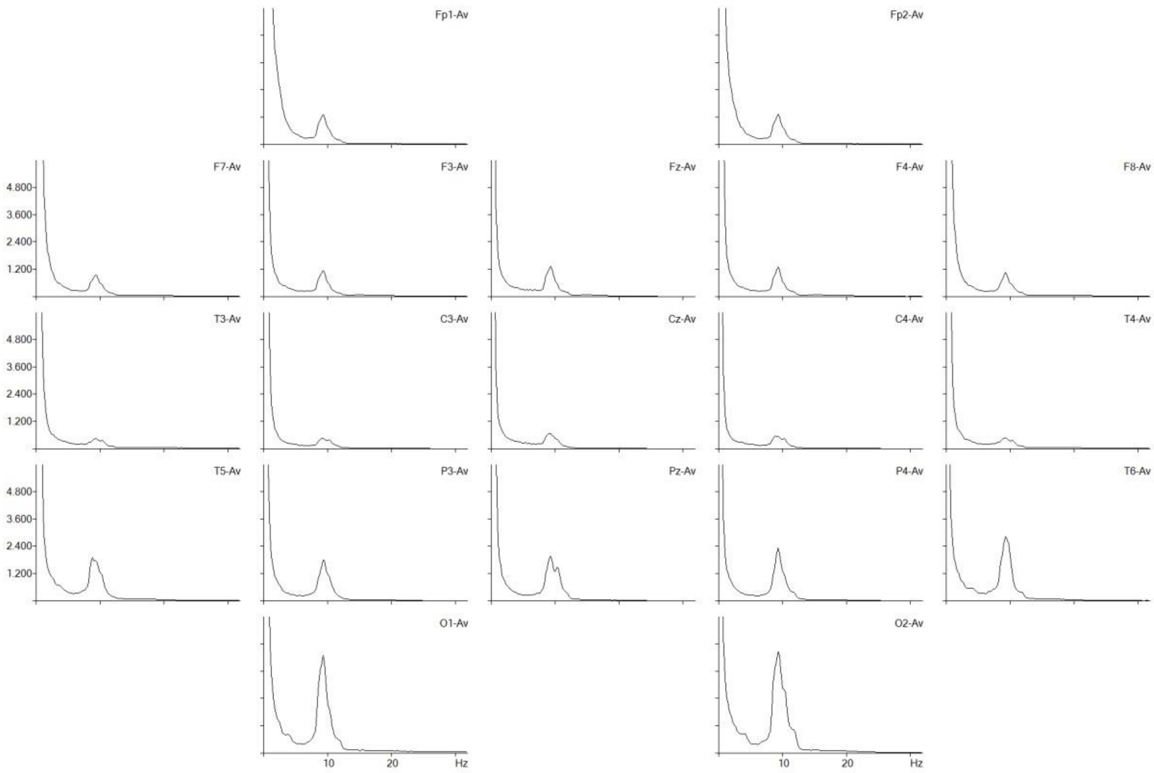

Maps of EEG power spectra for bandranges

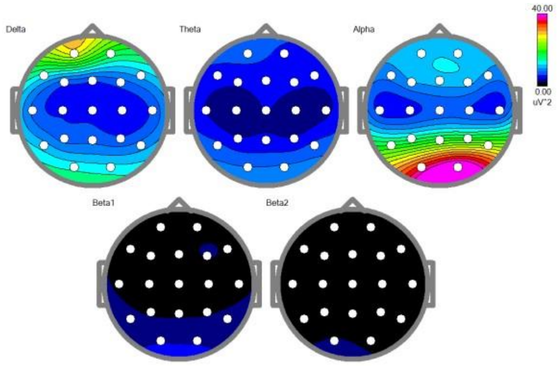

Graphs of EEG absolute power spectra comparison with HBI Database (14-15 yo)

Fragment: Grand Average, Offset: 0.00 s, Length: 314.08 s, Number of epochs 1.

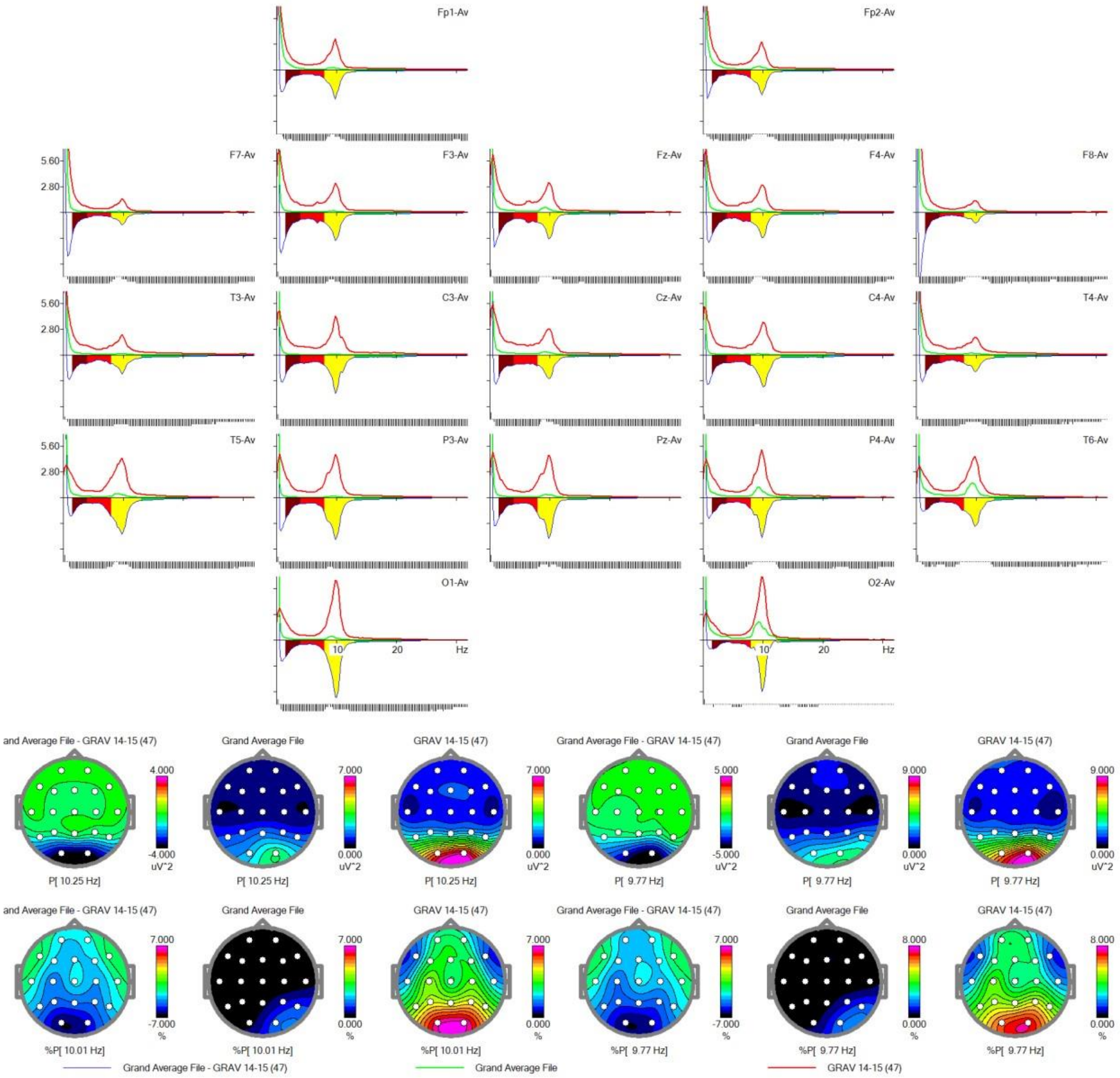

Trauma Group (TG) – Eyes Open Condition

25 participants with Complex Childhood Trauma (CCT) of which only 18 recordings included in grand average (enough de-artefacted epochs)

Graphs and Maps of EEG absolute power spectra comparison with HBI Database (14-15 yo)

Fragment: Grand Average, Offset: 0.00 s, Length: 311.34 s, Number of epochs 1.

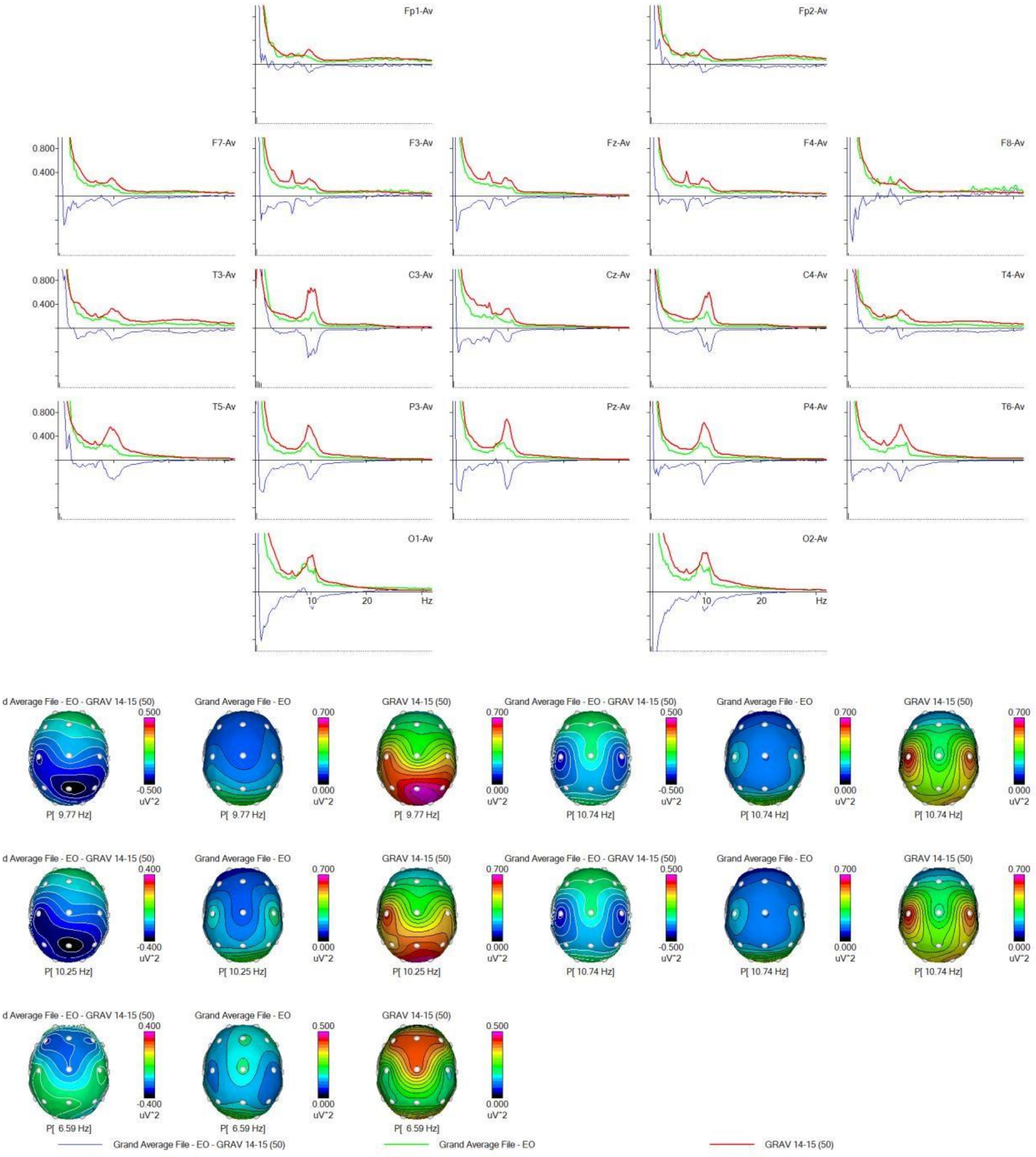

Note: Green line= Trauma Group (TG), Red line = Database, Blue line = Difference

Lateral Maps of EEG absolute power spectra comparison with HBI Database (14-15 yo) – right temporal area

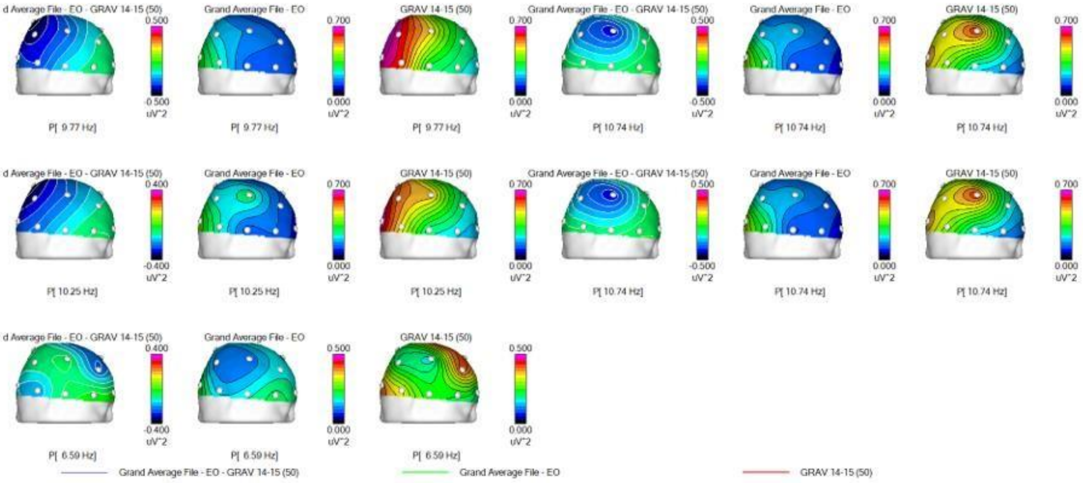

Note: Green line= Trauma Group (TG), Red line = Database, Blue line = Difference
