# Supplementary material for: Temporal–Posterior Alpha Power in Resting-State Electroencephalography as a Potential Marker of Complex Childhood Trauma in Institutionalized Adolescents
Source: Brain Sci. 2024 Jun 6;14(6):584. doi: 10.3390/brainsci14060584 (PMC11201643; doi:10.3390/brainsci14060584)
Supplement: Supplementary file 1 [file brainsci-14-00584-s001.zip › Table S1 Sample Descriptives.pdf]

Table 1 Sample descriptives

Demographic information and trauma assessment scores

Group	Half-siblings		Twins	
ID	S1	S2	T1	T2
Age (years)	13	17	15	15
Sex	female	female	female	male
IQ	92	72	85	100
ACEs (Adverse Childhood Experiences)	6	6	11	10
PTSD (Post-traumatic stress disorder)	6	14	10	14
DSO (Disturbances in Self-Organization)	0	3	12	12
CPTSD (Complex Post-traumatic stress disorder)	6	17	22	26
Subjective Symptoms Evaluation	21	38	29	35
